# Association between community socioeconomic factors, animal feeding operations, and campylobacteriosis incidence rates: Foodborne Diseases Active Surveillance Network (FoodNet), 2004–2010

**DOI:** 10.1186/s12879-016-1686-9

**Published:** 2016-07-22

**Authors:** Rachel E. Rosenberg Goldstein, Raul Cruz-Cano, Chengsheng Jiang, Amanda Palmer, David Blythe, Patricia Ryan, Brenna Hogan, Benjamin White, John R. Dunn, Tanya Libby, Melissa Tobin-D’Angelo, Jennifer Y. Huang, Suzanne McGuire, Karen Scherzinger, Mei-Ling Ting Lee, Amy R. Sapkota

**Affiliations:** Maryland Institute for Applied Environmental Health, University of Maryland School of Public Health, College Park, School of Public Health Building (255), 4200 Valley Drive, Room 2234P, College Park, MD 20742 USA; Department of Epidemiology and Biostatistics, University of Maryland School of Public Health, College Park, MD USA; Prevention and Health Promotion Administration, Maryland Department of Health and Mental Hygiene, Baltimore, MD USA; Emerging Infections Program, Disease Control and Environmental Epidemiology Division, Colorado Department of Public Health and Environment, Denver, CO USA; Communicable and Environmental Disease Surveillance, Tennessee Department of Health, Nashville, TN USA; California Emerging Infections Program, Oakland, CA USA; Acute Disease Epidemiology Section, Georgia Department of Public Health, Atlanta, GA USA; Office of Infectious Disease, National Center for Emerging and Zoonotic Infectious Diseases, Centers for Disease Control and Prevention, Atlanta, GA USA; New York State Department of Health, Albany, NY USA; New Mexico Emerging Infections Program, University of New Mexico, Albuquerque, NM USA

**Keywords:** *Campylobacter*, FoodNet, Community socioeconomic and environmental factors, Broiler chicken, Dairy

## Abstract

**Background:**

*Campylobacter* is a leading cause of foodborne illness in the United States. *Campylobacter* infections have been associated with individual risk factors, such as the consumption of poultry and raw milk. Recently, a Maryland-based study identified community socioeconomic and environmental factors that are also associated with campylobacteriosis rates. However, no previous studies have evaluated the association between community risk factors and campylobacteriosis rates across multiple U.S. states.

**Methods:**

We obtained *Campylobacter* case data (2004–2010; *n* = 40,768) from the Foodborne Diseases Active Surveillance Network (FoodNet) and socioeconomic and environmental data from the 2010 Census of Population and Housing, the 2011 American Community Survey, and the 2007 U.S. Census of Agriculture. We linked data by zip code and derived incidence rate ratios using negative binomial regression models.

**Results:**

Community socioeconomic and environmental factors were associated with both lower and higher campylobacteriosis rates. Zip codes with higher percentages of African Americans had lower rates of campylobacteriosis (incidence rate ratio [IRR]) = 0.972; 95 % confidence interval (CI) = 0.970,0.974). In Georgia, Maryland, and Tennessee, three leading broiler chicken producing states, zip codes with broiler operations had incidence rates that were 22 % (IRR = 1.22; 95 % CI = 1.03,1.43), 16 % (IRR = 1.16; 95 % CI = 0.99,1.37), and 35 % (IRR = 1.35; 95 % CI = 1.18,1.53) higher, respectively, than those of zip codes without broiler operations. In Minnesota and New York FoodNet counties, two top dairy producing areas, zip codes with dairy operations had significantly higher campylobacteriosis incidence rates (IRR = 1.37; 95 % CI = 1.22, 1.55; IRR = 1.19; 95 % CI = 1.04,1.36).

**Conclusions:**

Community socioeconomic and environmental factors are important to consider when evaluating the relationship between possible risk factors and *Campylobacter* infection.

## Background

An estimated 9.4 million domestically acquired foodborne illnesses, associated with 31 known pathogens, occur each year in the United States [1]. *Campylobacter*, a leading bacterial cause of these foodborne illnesses, is responsible for an estimated 1 million cases each year [1]. Campylobacteriosis is typically characterized by gastroenteritis [2]. More severe *Campylobacter* infections can lead to septicemia, arthritis, Guillain-Barré syndrome, or Miller Fisher syndrome [2]. *Campylobacter* normally inhabit the intestines of warm-blooded wild and domestic animals and several avian species [3]. Along with international travel, ingestion and handling of poultry and ingestion of dairy products contaminated with *Campylobacter* have been identified as major risk factors for both sporadic cases and outbreaks [2, 4–7]. Current interventions to reduce the incidence of *Campylobacter* infection in the United States have focused on improving food safety by the development of poultry industry performance standards; yet, incidence rates in 2013 were higher than in 2006–2008 [8, 9].

Beyond food-related risk factors, multiple analyses show that community socioeconomic and environmental risk factors, such as living in areas with higher median household incomes or living in close contact with livestock, influence the risk of campylobacterosis [7, 10, 11]. *Campylobacter* can enter the environment through direct fecal contamination of water bodies, manure application on agricultural land, and runoff [12, 13]. *Campylobacter* has been found in surface water and groundwater, and living in homes on private wells or ingesting water from lakes or rivers has been associated with an increased risk of campylobacteriosis [12–15].

A recent Maryland-based study evaluated the association between community socioeconomic and environmental risk factors and rates of campylobacteriosis and found that several factors, including degree of rurality and the presence of broiler chicken operations, were associated with campylobacteriosis rates [11]. However, campylobacteriosis rates differ by geographic region, and therefore, it is unknown whether Maryland-based findings can be extrapolated to other states [16]. Identifying whether community socioeconomic and environmental factors are associated with campylobacterosis across the U.S. is an important step towards improving our understanding of exposures associated with campylobacteriosis. This study investigated the association between zip code level socioeconomic and environmental variables and campylobacteriosis incidence at multiple surveillance sites in the U.S.

## Methods

### Data sources

The Foodborne Diseases Active Surveillance Network (FoodNet) is a collaboration between the Centers for Disease Control and Prevention (CDC), 10 state health departments, the US Department of Agriculture’s Food Safety and Inspection Service (USDA-FSIS), and the US Food and Drug Administration (FDA). The FoodNet surveillance area includes the states of Connecticut (CT), Georgia (GA), Maryland (MD), Minnesota (MN), New Mexico (NM), Oregon (OR), and Tennessee (TN), and selected counties in California (CA), Colorado (CO), and New York (NY). FoodNet conducts active population-based surveillance for laboratory-confirmed infections caused by nine pathogens transmitted commonly through food, including *Campylobacter*. For this study, we restricted analyses to data on culture-confirmed cases of *Campylobacter* infection (including infections caused by *C. jejuni, C. coli* and unknown *Campylobacter* spp.) reported between 2004 and 2010. Both sporadic cases and those associated with outbreaks were included in this analysis.

We obtained socioeconomic data from the 2010 Census of Population and Housing and the 2011 American Community Survey (5-year estimates) by 5-digit zip code tabulation area (ZCTA) (9). We selected socioeconomic variables on the basis of recommendations from studies conducted by Zappe Pasturel et al. [11] and Krieger et al. [17]. We obtained animal feeding operation data from the 2007 U.S. Census of Agriculture, National Agricultural Statistics Service [18].

### Descriptive analyses

We calculated *Campylobacter* incidence rates per 100,000 population by year for each state using intercensal estimates of state populations from the U.S. Census Bureau [19]. FoodNet *Campylobacter* case count data were linked with the socioeconomic and animal feeding operation data by zip code and 5-digit ZCTA and used to calculate rates per 100,000 population by zip code using zip code population estimates from the 2010 Census.

### Negative-binomial regression

We developed regression models to evaluate associations between socioeconomic and environmental factors and campylobacteriosis incidence at the zip code level. First, we evaluated collinearity among our predictor variables using the inverse of the variance inflation factor. Highly collinear variables were excluded using a stepwise approach. We compared several regression models for count data and tested models with and without zero inflation and with and without spatial covariate structure. The negative binomial regression model without spatial covariate structure provided the best fit for the dataset and the final model included the zip code level variables described in Table 1. Cases from Georgia between 2004 and 2008 (*n* = 3,112, 7.5 % of all campylobacteriosis cases reported to FoodNet between 2004 and 2010) and 762 cases (1.9 % of all reported cases) from the other FoodNet sites were excluded from the final model because either zip code information was missing or socioeconomic Census variables were not available for the given zip code. A total of 4,692 zip codes were included in the analysis. The number of zip codes included per state was as follows: CA, 116; CO, 133; CT, 271; GA, 710; MD, 450; MN, 880; NM, 345; NY, 757; OR, 413; TN, 617. We ran both a regression model that included all FoodNet sites and site-specific regression models. We performed all modeling using SAS version 9.3, and used *p*-values of ≤0.05 to assess statistical significance.

**Table 1:** Sources and files for the socioeconomic and environmental variables included in the negative binomial regression models used in this study

Socioeconomic variable	Data source	File
1. Percentage of housing units in rural areas, on a scale of 0 % to 100 %	2010 Census	SF1 H2
2. Presence or absence of broiler chicken operations	2007 U.S. Census of Agriculture, National Agricultural Statistics Service	
3. Presence or absence of dairy operations	2007 U.S. Census of Agriculture, National Agricultural Statistics Service	
4. Presence or absence of turkey operations	2007 U.S. Census of Agriculture, National Agricultural Statistics Service	
5. Median age	2010 Census	Summary File 1 (SF1) DP1
6. Percentage of the population composed of African Americans	2010 Census	SF1 DP1
7. Percentage of the population composed of Hispanics	2010 Census	SF1 DP1
8. Percentage of owner-occupied housing units	2010 Census	SF1 DP1
9. Percentage of the population aged 25 years and older without a high school diploma	2011 American Community Survey	DP03 Report
10. Percentage of individuals living below the poverty level	2011 American Community Survey	DP03 Report

## Results

From 2004 to 2010, 40,768 cases of culture-confirmed *Campylobacter* infection were reported to FoodNet. Of those cases, 36,894 had valid zip codes, for which Census data were available, and were included in subsequent analyses. About 45 % of cases were confirmed as *C. jejuni*, 2.4 % as *C. coli* and 52.7 % as unknown *Campylobacter* spp. Most cases were sporadic infections (99.6 %), while 0.4 % of cases were associated with outbreaks. Most cases were white (62.4 %), 3.8 % were African-American, 3.3 % were Asian, and 30.5 % were of other or unknown race. In terms of age, 12.6 % of cases were 0–4 years, 5.2 % were 5–9 years, 9.5 % were 10–19 years, 56.6 % were 20–59 years, and 16.1 % were ≥60 years.

**Fig. 1:**
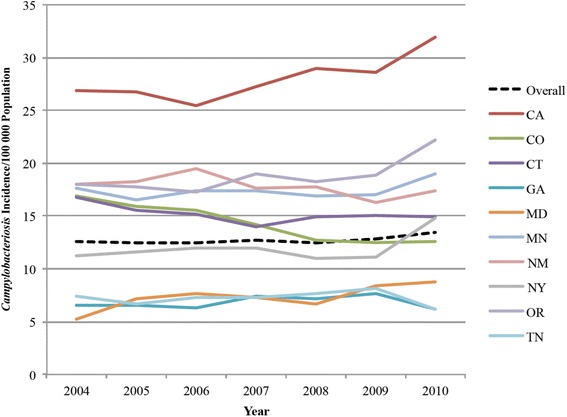
Incidence of *Campylobacter* infection per 100,000 population by year and by FoodNet site: 10 FoodNet sites, 2004–2010

The average annual incidence of campylobacteriosis across all 10 FoodNet sites ranged from 12.4 per 100,000 population in 2005 to 13.4 per 100,000 population in 2010 (Fig. 1). California had the highest average incidence (28.0 cases per 100,000 population) and Georgia had the lowest (6.8). The highest incidence among all sites was in California in 2010 (32.0), while the lowest was in Maryland in 2004 (5.2).

### Community socioeconomic factors

The overall regression model identified multiple socioeconomic factors at the zip code level that were associated with campylobacteriosis incidence rates. Incidence was higher in zip codes with higher percentages of Hispanic residents (incidence rate ratio [IRR] = 1.005; 95 % confidence interval [CI] = 1.002,1.007); and in zip codes with higher percentages of individuals living below the poverty level (IRR = 1.011; 95 % CI = 1.006,1.015) (Table 2). Incidence was lower in zip codes with higher percentages of African American residents (IRR = 0.972; 95 % CI = 0.97,0.974), higher owner occupancy rates (IRR = 0.995; 95 % CI = 0.992,0.997), and higher percentages of the population aged 25 years and older without a high school diploma (IRR = 0.967; 95 % CI = 0.96,0.973) (Table 2).

**Table 2:** Campylobacteriosis in association with community environmental and socioeconomic factors: 10 FoodNet sites, 2004–2010

Zip Code Variable	Negative Binomial Regression IRR (95 % CI)										
	Overall	CA	CO	CT	GA	MD	MN	NM	NY	OR	TN
% Rurality	1.002 (1.001, 1.003)	1.012 (1.005, 1.02)	0.998 (0.993, 1.002)	1.001 (0.999, 1.003)	1.002 (0.999, 1.005)	1.00 (0.998, 1.002)	1.005 (1.003, 1.006)	1.00 (0.997, 1.004)	1.004 (1.003, 1.006)	1.005 (1.003, 1.007)	1.001 (0.998, 1.003)
Broiler chicken operations	0.867 (0.806, 0.932)	1.00 (1.00, 1.00)	1.021 (0.663, 1.572)	1.007 (0.818, 1.239)	1.216 (1.031, 1.433)	1.163 (0.987, 1.369)	1.046 (0.939, 1.165)	0.841 (0.473, 1.496)	0.98 (0.841, 1.141)	1.074 (0.941, 1.227)	1.346 (1.184, 1.53)
Dairy operations	1.045 (0.977, 1.119)	1.192 (0.77, 1.846)	0.356 (0.234, 0.54)	0.794 (0.696, 0.906)	0.92 (0.784, 1.08)	0.966 (0.83, 1.123)	1.372 (1.216, 1.548)	1.028 (0.778, 1.359)	1.189 (1.037, 1.363)	1.018 (0.896, 1.155)	1.114 (0.982, 1.264)
Turkey operations	1.129 (1.033, 1.234)	0.507 (0.237, 1.082)	0.835 (0.587, 1.189)	0.808 (0.67, 0.976)	0.795 (0.536, 1.177)	0.776 (0.627, 0.961)	1.016 (0.911, 1.133)	1.112 (0.739, 1.671)	0.963 (0.817, 1.136)	0.989 (0.855, 1.144)	0.872 (0.734, 1.035)
Median age, years	1.00 (0.994, 1.006)	1.029 (1.014, 1.044)	0.989 (0.961, 1.018)	1.037 (1.019, 1.055)	1.017 (0.996, 1.038)	1.026 (1.01, 1.042)	1.007 (0.998, 1.017)	0.953 (0.935, 0.972)	0.983 (0.969, 0.997)	0.988 (0.976, 1.001)	1.023 (1.007, 1.039)
% African American population	0.972 (0.97, 0.974)	0.991 (0.983, 0.999)	0.98 (0.966, 0.999)	0.997 (0.99, 1.003)	0.986 (0.981, 0.99)	0.985 (0.982, 0.988)	1.007 (0.996, 1.017)	0.917 (0.817, 1.03)	0.988 (0.981, 0.995)	1.018 (0.995, 1.041)	0.993 (0.989, 0.997)
% Hispanic population	1.005 (1.002, 1.007)	0.999 (0.991, 1.007)	0.982 (0.966, 0.999)	1.008 (1.00, 1.016)	1.004 (0.995, 1.013)	0.997 (0.99, 1.005)	1.018 (1.007, 1.03)	0.997 (0.992, 1.001)	0.994 (0.976, 1.012)	1.00 (0.992, 1.008)	0.992 (0.974, 1.01)
% Owner occupancy	0.995 (0.992, 0.997)	0.988 (0.983, 0.992)	1.002 (0.992, 1.012)	0.992 (0.985, 0.998)	0.99 (0.983, 0.997)	0.996 (0.99, 1.002)	0.994 (0.989, 1.00)	1.018 (1.006, 1.031)	0.999 (0.992, 1.005)	0.998 (0.991, 1.005)	0.998 (0.991, 1.006)
% Population aged ≥ 25 years without a high school diploma	0.967 (0.96, 0.973)	0.985 (0.955, 1.015)	1.002 (0.953, 1.053)	0.934 (0.91, 0.958)	0.995 (0.976, 1.014)	0.984 (0.967, 1.001)	0.97 (0.95, 0.992)	1.006 (0.986, 1.026)	0.991 (0.974, 1.008)	0.961 (0.943, 0.978)	0.998 (0.982, 1.015)
% Residents below poverty level	1.011 (1.006, 1.015)	0.996 (0.981, 1.012)	1.03 (1.002, 1.058)	0.999 (0.98, 1.018)	1.008 (0.995, 1.021)	1.019 (1.006,1.032)	0.989 (0.979, 1.00)	1.021 (1.01, 1.033)	0.998 (0.988, 1.008)	0.998 (0.989, 1.007)	1.01 (1.001, 1.019)

When stratified by FoodNet site, the direction of the association and statistical significance for each socioeconomic variable varied, but we observed some common patterns (Table 2). In six sites (CA, CO, GA, MD, NY, and TN), zip codes characterized by higher percentages of African Americans had significantly lower rates of campylobacteriosis (Table 2). In four sites (CO, MD, NM, and TN), zip codes that had a higher percentage of individuals living below the poverty level had higher incidence rates (Table 2). Higher owner occupancy rate was inversely associated with *Campylobacter* infections in four sites (CA, CT, GA, and MN); however, the direction of the association was reversed in New Mexico (Table 2).

### Community environmental factors

Several environmental factors at the zip code level were associated with campylobacteriosis incidence in the overall model. Incidence was slightly higher in zip codes with higher percentages of housing units within rural areas (IRR = 1.002; 95 % CI = 1.001,1.003); and in zip codes with turkey operations (IRR = 1.129; 95 % CI = 1.033,1.234). Incidence was lower in zip codes with broiler operations (IRR = 0.867; 95 % CI = 0. 806, 0.932).

**Fig. 2:**
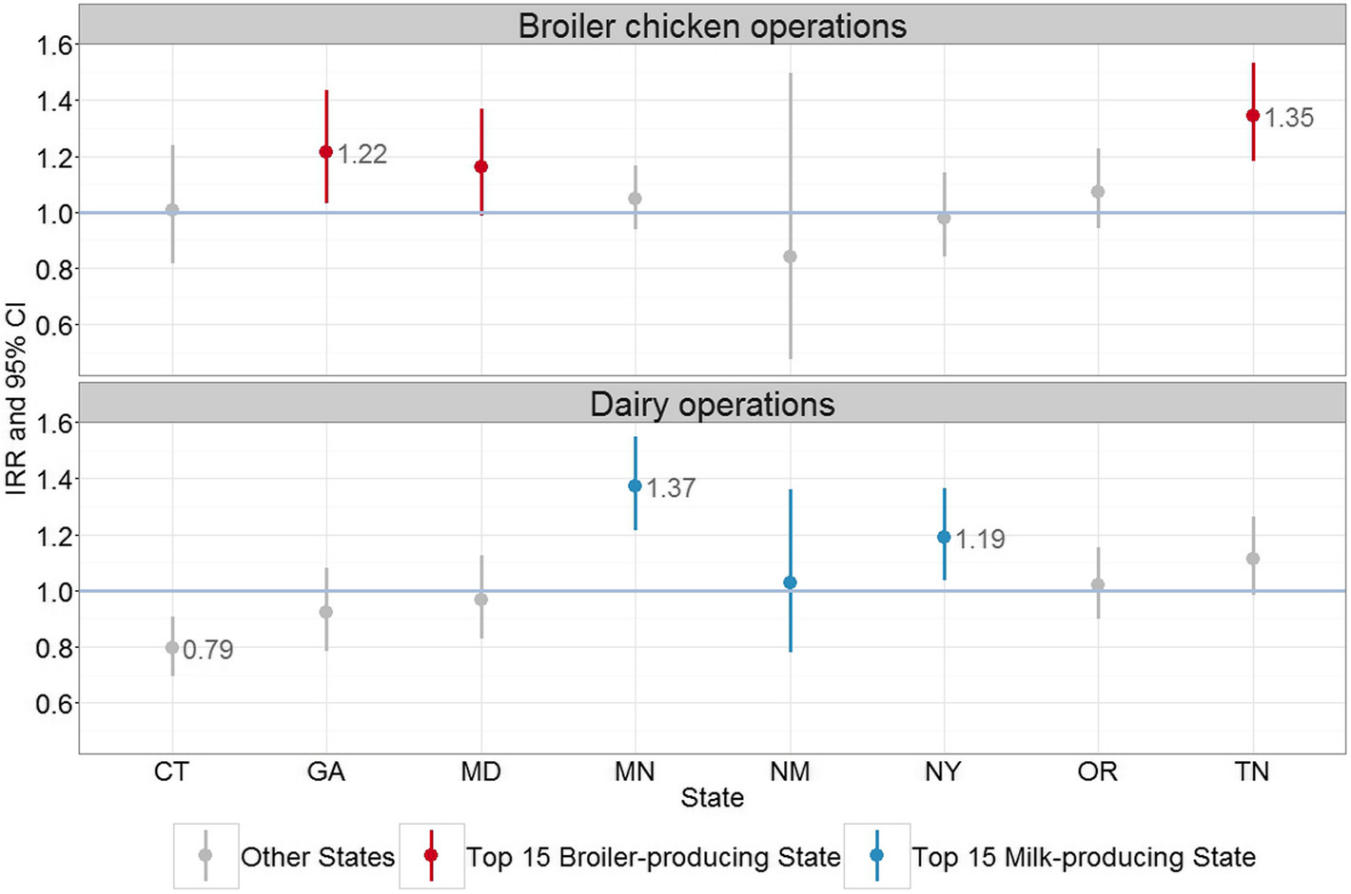
*Campylobacter* incidence rate ratios for the presence of broiler chicken and dairy operations in a zip code at 8 FoodNet sites. The FoodNet catchment areas for California and Colorado predominantly include more urban areas, are not representative of the entire state, and were not included in the figure

When stratified by FoodNet site, we observed some interesting patterns for environmental factors (Table 2). In four sites (CA, MN, NY, and OR), zip codes with higher percentages of housing units in rural areas had higher incidence rates of campylobacterosis. In Georgia and Tennessee, zip codes with broiler operations had significantly higher incidence rates than those without. In Georgia, incidence was 22 % higher in zip codes with broiler operations (IRR = 1.22, 95 % CI = 1.03,1.43), and in Tennessee, incidence was 35 % higher in zip codes with broiler operations (IRR = 1.35, 95 % CI = 1.18,1.53) (Fig. 2). The findings were similar in Maryland, but the association was not significant (IRR = 1.163, 95 % CI = 0.99,1.37) (Fig. 2).

In Minnesota and New York FoodNet counties, incidence was 37 % higher (IRR = 1.37; 95 % CI = 1.22,1.55) and 19 % higher (IRR = 1.19; 95 % CI = 1.04,1.36), respectively, in zip codes with dairy operations (IRR = 1.37; 95 % CI = 1.22,1.55) (Fig. 2). In contrast, in the Colorado FoodNet counties and Connecticut, the incidence of campylobacteriosis was lower in zip codes with dairy operations compared to those without dairies (IRR = 0.36, 95 % CI = 0.23,0.54; IRR = 0.79, 95 % CI = 0.70,0.91, respectively).

## Discussion

To our knowledge, this is the first study to evaluate the effect of community socioeconomic and environmental factors on *Campylobacter* incidence across multiple U.S. sites by linking surveillance data with publicly available data sources at the zip code level. Our results emphasize the importance of evaluating community risk factors for differing sites individually, because analyzing the data overall obscured some patterns.

### Community socioeconomic factors

In six of the 10 FoodNet sites, campylobacteriosis incidence rates were lower in zip codes with higher percentages of African Americans. This finding corroborates previous findings in Maryland, as well as findings from Samuel et al. [20] that included data from all FoodNet sites [11]. These findings might be influenced by differences in healthcare access among different races and ethnicities. African Americans are less likely to have health insurance and are more likely to have structural impediments to healthcare which decreases the opportunities for infections among this population to be captured by surveillance programs [21]. Quinlan et al. [22] also suggested that African Americans could have greater immunity to *Campylobacter*.

In our overall regression model and in two of the individual FoodNet sites, campylobacteriosis incidence rates were higher in zip codes with higher percentages of Hispanics. Two recent studies analyzing risk factors for *Campylobacter* infections found similar associations between Hispanic ethnicity and increased risk of campylobacteriosis, including a telephone survey among residents in the 10 FoodNet sites [23, 24]. Neither of these studies posited why Hispanic ethnicity was associated with a higher risk of campylobacteriosis, and research concerning this association is still lacking.

Our results also showed that some zip codes characterized by lower socioeconomic status had higher incidence rates of campylobacterosis, suggesting that poverty might be associated with higher rates of *Campylobacter* infection. However, our results for all variables associated with poverty do not clearly support this hypothesis, and the literature is similarly unclear about the association between poverty and campylobacterosis incidence. For instance, in our overall regression model, zip codes with more individuals living below the poverty level, as well as zip codes with lower owner occupancy rates, had higher campylobacteriosis rates. However, there were lower rates of campylobacterosis in zip codes with higher percentages of individuals without a high school diploma (another indicator of low socioeconomic status). Krieger et al. [25] found that poverty increased the risk of multiple negative health outcomes including bacterial infections. Darcey and Quinlan [26] evaluated the number of health code violations at food retail facilities in the Philadelphia, Pennsylvania area as a surrogate for foodborne illness and found that there were more critical health violations in food service facilities in high poverty areas [22]. Bemis et al. [27] also showed that, among children less than 10 years old, lower socioeconomic status was associated with a higher incidence of campylobacteriosis. These findings are particularly interesting since lower socioeconomic status has been associated with several barriers to healthcare access--including financial barriers and stigma associated with using Medicaid--that would seemingly reduce the probability of poorer individuals seeking healthcare and being captured by surveillance systems [28]. On the other hand, some studies have noted that higher socioeconomic status is associated with higher rates of campylobacteriosis [27].

### Community environmental factors

Several studies have suggested that environmental factors contribute more to the incidence of campylobacteriosis than previously thought [3, 22, 29]. The high variability of campylobacteriosis incidence across FoodNet sites supports the hypothesis that one’s environment might affect risk. Rurality was significantly associated with higher incidence rates of campylobacteriosis in our overall model as well as in site-specific regression models for four sites. The higher rate of *Campylobacter* infections in rural areas might be explained by an increased likelihood of exposure to animals and animal waste [11]. A previous study by Green et al. [30] identified a 1.46 higher campylobacteriosis incidence rate in rural areas of Manitoba, Canada compared to urban areas.

Poultry consumption is the most common risk factor for sporadic *Campylobacter* infections, and a leading risk factor for *Campylobacter* outbreaks, in the U.S. [4, 31]. Approximately 90 % of U.S. chicken flocks are colonized with *Campylobacter* [29]. Georgia and Tennessee, two of the leading broiler producing states in the U.S., had significantly higher incidence rates of campylobacteriosis (22 % and 35 % higher, respectively) in zip codes containing broiler chicken operations compared to zip codes without these operations (Table 2) [32]. Our results for Maryland, another leading broiler producing state, also suggest a relationship between the presence of broiler operations in a zip code and higher campylobacteriosis incidence rates.

*Campylobacter* could be spread from broiler operations to human populations through both surface water and groundwater. Broiler chickens shed *Campylobacter* in their feces, and it could then enter nearby surface waters after land application of poultry litter and during runoff events [33]. *Campylobacter* has been isolated from several types of surface water, including streams and rivers, and can survive in water for at least 29 days [29, 34]. Wilkes et al. [13] detected *Campylobacter* in Canadian surface water samples more frequently during cooler seasons when poultry litter is more typically applied to land. Vereen et al. [35] found more *Campylobacter* in streams downstream of poultry houses, as well as a positive association between the frequency of detecting *Campylobacter* and the number of poultry houses in a subwatershed. Contaminated groundwater is also a possible source of *Campylobacter* exposure among humans. The majority of *Campylobacter* outbreaks attributed to drinking water between 1997 and 2008 in the U.S. were associated with untreated groundwater [4].

Cows and other ruminants also have been identified as important sources of *Campylobacter* infection in both the U.S. and Europe [3, 4]. Taylor et al. [4] identified that the consumption of dairy products was the largest contributor (28.9 %) to U.S. campylobacteriosis outbreaks from 1997 to 2008. *Campylobacter* could be transmitted through the environment from dairy operations to human populations through water from direct fecal contamination of water bodies or from land application of dairy waste. A study in northwest England found that *Campylobacter* concentrations, specifically *C. jejuni*, increased as water flowed downstream through dairy grazing pastures [36]. When dairy wastewater is land applied, *Campylobacter* can also become airborne and could infect individuals downwind of dairy wastewater application sites [37, 38].

Our data support the findings of Arsenault et al. [3] in Quebec, who reported that higher density of ruminants was significantly associated with increased incidence of campylobacteriosis, especially among children. In New York FoodNet counties and Minnesota, two leading dairy producing regions [39], zip codes containing dairy operations had statistically significantly higher incidence rates of campylobacterosis compared to zip codes without dairies. In California and Colorado, two other important dairy producing states in the U.S., we could not effectively evaluate this relationship because the FoodNet catchment areas associated with these sites do not include the full states.

The type of dairy operation (e.g., concentrated animal feeding operation (CAFO), free range, conventional, organic), could also impact the risk of *Campylobacter* infection in a zip code. A study by Rapp et al. [14] found that dairy cows at CAFOs were more likely to shed *Campylobacter* than dairy cows at free range operations in New Zealand. A study conducted in the Midwestern and northeastern regions of the U.S. found that conventional dairy operations had more *Campylobacter-*positive fecal and environmental samples, and higher proportions of antibiotic-resistant *Campylobacter* isolates, compared to organic farms [40]. However, a similar study by Sato et al. [41] found no statistically significant differences in *Campylobacter* presence or antimicrobial resistance between conventional and organic dairy farms. The impact of the type of dairy operation on incidence of *Campylobacter* infections was not the focus of the current study, but deserves further attention.

### Limitations

Our study had some limitations. First, because the FoodNet sites in California, Colorado, and New York do not cover the entire state but only select counties we could not fully examine some factors of interest at those sites. Second, the data collected by FoodNet is limited to specific U.S. sites and might not be representative of the whole country. In addition, since we engaged in an ecological study design, providing data on the association between community-level risk factors and campylobacteriosis incidence, our findings cannot be used to infer associations at the individual level. Finally, because the FoodNet data available to us was at the zip code level, the level of resolution of our analyses is not as fine as it would have been had the data been available at the census block or census tract level.

## Conclusions

Our findings point to several community socioeconomic and environmental factors that might be associated with campylobacteriosis incidence. The increased incidence rates in zip codes containing broiler and dairy operations in states that are leading producers in those industries, as well as the inverse association in zip codes with more African Americans, were of interest. Previous social epidemiological studies have shown that community-level factors are important with regard to a range of diseases and health outcomes. Our data are among the first to show that it is important to consider both community-level socioeconomic and environmental factors, in addition to individual-level factors, when examining risk factors for *Campylobacter* infection. The unique patterns of association observed at individual sites emphasize the importance of analyzing smaller areas when investigating the association between socioeconomic and environmental factors and campylobacteriosis incidence. The heterogeneity of our findings across sites suggests that future studies of this nature may benefit from the inclusion of both individual-level and community-level factors in the modeling approach.

## Abbreviations

95 % CI, 95 % confidence interval; CA, California; CDC, centers for disease control and prevention; CAFO, concentrated animal feeding operation; CT, connecticut, CO, Colorado; FDA, Food and Drug Administration; FoodNet, Foodborne Diseases Active Surveillance Network; GA, Georgia; IRR, Incidence rate ratio; MD, Maryland; MN, Minnesota; NM, New Mexico; NY, New York; OR, Oregon; TN, Tennessee; USDA-FSIS, US Department of Agriculture, Food Safety and Inspection Service; ZCTA, Zip code tabulation area.
